# Behavioral gender differences across Pre-School Children with Autism Spectrum Disorders: a cross-sectional study

**DOI:** 10.1007/s10803-022-05498-y

**Published:** 2022-03-15

**Authors:** Marco Tofani, Lucia Scarcella, Giovanni Galeoto, Federica Giovannone, Carla Sogos

**Affiliations:** 1 Department of Intensive Neurorehabilitation and Robotics, Bambino Gesù Children Hospital, IRCCS, Rome, Italy; 2 Department of Human Neurosciences, Sapienza University of Rome, Rome, Italy

**Keywords:** Autism spectrum disorder, Gender, Behavior, Pre-school, Sensory interest, Verbal Communication

## Abstract

There is increasing literature showing that the presentation of Autism Spectrum Disorder (ASD) could be different according to the sex of the patient. Through the analysis of the Autism Diagnostic Interview Revised interview results of a study group consisting of 56 preschool children diagnosed with ASD potential differences in the presentation of ASD were searched. Variables investigated were verbal abilities, the presence/absence of unusual sensory interests, as well as of echolalia. The results showed significant differences between gender in restricted and repetitive behavior (p < 0.01), in particular for those children who have no unusual sensory interest (p < 0.05) and with minimal verbal ability (p < 0.05). The findings contribute providing evidences on phenotypical differences in preschool children with a diagnosis of Autism Spectrum Disorder.

**Introduction**.

Autism spectrum disorder (ASD), a constellation of neurodevelopmental conditions with heterogeneous etiologies, has been reported to be more prevalent in males since the initial case series (Lai et al., 2014). It is important to state that “sex” refers to “the biological and physiological characteristics that define men and women” and that “gender” refers to “the socially constructed roles, behaviors, activities, and attributes that a given society considers appropriate for men and women” (WHO, 2021). Since most human studies of autism focus on children, adolescents, and adults, it is difficult to separate the effect of sex and gender since gendered socialization begins at birth (Lai et al., 2015). For this reason, the term “sex/gender” was used to acknowledge the inevitable overlap between the two terms.

The reasons for sex/gender differences have been investigated by numerous researchers, with many claiming that sex/gender discrepancies are lower than currently thought (Kim et al., 2011; Mattila et al., 2011). However, sex/gender differences in ASD have been observed at an early age: females with ASD showed relative strengths in joint attention, but impairments in imitation at 18 months of age (Øien et al., 2017). This might indicate that less severe cases in females are not identified and consequently do not receive an ASD diagnosis. This is in line with previous studies (Dworzynski et al., 2012; Robinson et al., 2013) that suggest that females need to demonstrate more severe developmental, behavioral, or intellectual delay/deviance to be diagnosed with ASD. Another hypothesis is that the difference in sex ratio and symptom pattern might be related to the fact that males tend to show a higher level of repetitive behavior than females (Szatmari et al., 2012), while females tend to express better and more complex language than males (Salomone et al., 2016). According to Gould and Smith (Gould, 2017), girls were able to recognize themselves as being different from their peers and subsequently camouflaged it by mimicking speech and behaviors of other children of the same gender, but without feeling emotionally connected to them. Moreover, in a study by Milner et al. (Milner et al., 2019), most of the female participants with ASD reported camouflaging and masking their difficulties.

Regarding social interaction and communication, the literature is not consistent. According to various studies, females on average present fewer deficits in these domains compared to their male counterparts (Head et al., 2014; Hiller et al., 2014; Rynkiewicz et al., 2016). Conversely, other studies found worse social skills in girls (Hartley and Sikora, 2009; Lawson et al., 2018). Moreover, while a recent study by Ros-Demarize et al. (2020) reported that females demonstrated greater deficits in social communication, with similar results in both parental reports and standardized observations, a previous study by Mandy et al. (Mandy et al., 2012) showed differences in how parents and teachers, who are often the first to recognize potential autistic traits, evaluate social skills. In the latter research, while parents reported an equivalent impairment in social and communication areas in both sexes/genders, teachers more often recognized social difficulties in males since they showed more externalizing problems than females. It is not uncommon, especially among parents or caretakers, to perceive certain behaviors differently when performed by a young boy or girl. Characteristics such as shyness or oversensitivity, for example, are sometimes seen as typical traits for females in general, so they may be overlooked in girls (Skuse et al., 2004), potentially leading to under or late recognition of autistic traits in young females.

Regarding restricted and repetitive interests, an increasing number of studies and publications show that their prevalence is lower in females with ASD with respect to males with ASD. According to Rubenstein’s review of articles addressing gender differences in ASD (Rubenstein et al., 2015), literature shows that restricted interests occur more frequently in males independent of cognitive ability and that they are more prone to show routines and rituals. Moreover, in the previously cited study by Mandy et al. (Mandy et al., 2012), females presented less repetitive stereotyped behaviors, both through parental reports and direct observation. An increasing number of studies show differences in restricted and repetitive behaviors between females and males that are both qualitative, i.e., females tend to have fewer routines and limited restricted interests, and quantitative, i.e., these behaviors are less frequent in females. However, females with normal or mildly impaired intellectual abilities can camouflage their symptoms, so these patients must be evaluated carefully.

The aim of the present investigation was to explore sex/gender differences across preschool children with ASD, focusing on the three developmental domains evaluated in the Autism Diagnostic Interview- Revised (ADI-R) (Lord et al., 1994).

**Methods**.

**Participants and setting**.

The present cross-sectional study was conducted in the child psychiatry outpatient clinic of the Department of Human Neurosciences of Sapienza University of Rome, Italy. The research group was composed of medical doctors and rehabilitation professionals of the Department of Human Neurosciences, together with the Rehabilitation and Outcome Measures Assessment (ROMA) association. This research group has published diverse studies on ASD populations (Auletta et al., 2020; Catino et al., 2017; Cavalli et al., 2022; Pittella et al., 2018; Manti, Giovannone, & Sogos, 2019; Fioriello et al., 2020; Berardi et al., 2021; D’Alvia et al., 2020; Tofani et al., 2019).

Participants were children diagnosed with ASD, all under 6 years of age at the time of evaluation, with similar cognitive levels as assessed through cognitive examination. Recruitment was performed from March 2020 to December 2020. The variables investigated were sex/gender, verbal skills, echolalia, and unusual sensory interests. For ASD diagnosis, the ADI-R was used. Sex/gender differences in ADI-R scores across all variables were also investigated.

***Variables and analysis***.

Sample homogeneity was investigated with a non-parametric test for gender distribution, as well as for unusual sensory interest, echolalia, and verbal sub-group classification. To analyze differences in ADI-R scores between gender and each variable, a two-tailed independent sample t-test with a significance level of p ≤ 0.05 was used. The variables investigated were as follows:

***ADI-R.*** (Rutter, Le Couteur, & Lord, 2003.) This test was part of the diagnostic evaluation of participants. In this diagnostic tool, patient caregivers answer 93 questions regarding three main areas: social interaction (ADI-R A), communication and language (ADI-R B), and restricted and repetitive behaviors (ADI-R C). For the present investigation, the mean (standard deviation, SD) score of each sub-test was investigated and compared between males and females.

***Verbal skills.*** Individual verbal abilities of participants were assessed in the diagnostic evaluation through clinical observation. Based on the results, patients were classified as belonging to one of three groups: (1) non-verbal, those who were not able to produce any words, or were only able to say mom/dad; (2) minimally verbal, those who were able to say recognizable words but could not articulate sentences; or (3) verbal, those who were able to form sentences. Verbal skills were evaluated by an experienced medical doctor specialized in child neuropsychiatry.

***Echolalia and unusual sensory interests.*** Echolalia is a verbal disorder that consists of the repetition of another person’s speech (Kanner, 1946), either immediate or delayed. Unusual sensory interests are defined as atypical behaviors towards different sensory stimuli or sensory aspects in their surroundings (American Psychiatric Association, 2013). Both these characteristics are more often found in children with ASD. The presence or absence of these signs was assessed through clinical observation during the diagnostic evaluation.

**Results**.

**Participants**.

The total sample included 56 individuals: 44 males with a mean (SD) age of 36.41 months (12.6) and 12 females with a mean (SD) age of 36.08 months (7.6). The non-parametric test confirmed homogeneity for age (p = 0.93), gender (p = 0.64), echolalia (p = 0.64), verbal sub-group (0.19), and unusual sensory interest (p = 0.36) distribution. Statistically significant differences (p < 0.001) in ADI-R C scores (restricted and repetitive behaviors) were found, while no differences were found for other ADI-R subtests, namely social interaction and communication and language. Sample characteristics are summarized in Table 1.

**Table 1:** Sample characteristics

	Male	Female	Differences
Age in months, mean (SD)	36.41 (12.6)	36.08 (7.6)	0.93
Sample	44	12	0.64
Unusual sensory interests	25	5	0.36
Verbal sub-group	10	4	0.19
Echolalia	8	7	0.64
ADI-R A mean (SD) score	13.59 (6.56)	13.58 (5.08)	0.99
ADI-R B mean (SD) score	10.05 (4.09)	9.33 (4.39)	0.62
ADI-R C mean (SD) score	4.36 (2.93)	2.42 (1.62)	0.005*

***Differences in ADI-R scores across verbal subgroups***. Analyzing differences in ADI-R sub-test scores according to the verbal abilities of participants, sex/gender differences were found in ADI-R C scores in minimally verbal children (p = 0.012), while no differences were found in non-verbal (p = 0.35) or verbal (p = 0.10) children. In minimally verbal children, males had a higher mean score on the ADI-R C than females (males: 3.78 (SD 2.74); females = 2.00 (SD 0.70)). For both the ADI-R A and ADI-R B, no differences were found for any verbal sub-group, i.e., non-verbal, minimally verbal, or verbal. Data are summarized in Table 2.

**Table 2:** Differences in ADI-R scores across verbal sub-groups

	Mean (SD)	Mean (SD)	Significance
**Non verbal**	**Male n. 11**	**Female n. 3**	**Student-T**
ADI-R A	18.45 (5.46)	12.67 (3.05)	0.05
ADI-R B	11.27 (1.84)	13.33 (1.53)	0.12
ADI-R C	5.00 (2.65)	3.33 (2.31)	0.35
**Minimally verbal**	**Male n. 23**	**Female n. 5**	
ADI-R A	12.83 (5.99)	14.00 (5.01)	0.66
ADI-R B	9.91 (4.74)	8.20 (1.09)	0.13
ADI-R C	3.78 (2.74)	2.00 (0.70)	0.012*
**Verbal**	**Male n. 10**	**Female n. 4**	
ADI-R A	10.00 (6.23)	13.75 (7.27)	0.40
ADI-R B	9.00 (4.26)	7.75 (6.80)	0.75
ADI-R C	5.00 (3.59)	2.25 (2.06)	0.10

## Differences in ADI-R scores across echolalia sub-groups

Regarding qualitative data, females without echolalia reported higher scores in communication and language (ADI-R B) than males, while males with echolalia showed higher values for both communication and language (ADI-R B) and restricted and repetitive behaviors (ADI-R C). However, no statistically significant sex/gender differences were found in ADI-R scores in children with or without echolalia. Data are summarized in Table 3.

**Table 3:** ADI-R scores across echolalia sub-groups

	Mean (SD)	Mean (SD)	Significance
**No echolalia**	**Male n. 17**	**Female n. 5**	**Student-T**
ADI-R A	14.82 (6.91)	14.00 (2.82)	0.77
ADI-R B	9.76 (3.36)	13.50 (2.12)	0.18
ADI-R C	4.12 (2.39)	4.00 (2.83)	0.96
**Echolalia**	**Male n. 8**	**Female n. 7**	
ADI-R A	11.88 (4.22)	13.00 (5.29)	0.76
ADI-R B	10.00 (3.21)	8.33 (1.53)	0.28
ADI-R C	4.75 (4.03)	2.00 (1.00)	0.11

***Differences in ADI-R scores across unusual sensory interest sub-groups***: Sex/gender differences in ADI-R C scores were found in children with no unusual sensory interests (p = 0.017). Males had a higher mean score for restricted and repetitive behavior than females (males: 4.42 (SD 2.98); females: 2.14 (SD 1.46)). No sex/gender differences for any ADI-R sub-test were found in children with unusual sensory interests. Data are summarized in Table 4.

**Table 4:** ADI-R scores across unusual sensory interest sub-groups

	Mean (SD)	Mean (SD)	Significance
**No unusual sensory interest**	**Male n. 13**	**Female n. 7**	**Student-T**
ADI-R A	13.21 (7.09)	13.71 (6.05)	0.86
ADI-R B	10.32 (5.08)	8.57 (5.19)	0.46
ADI-R C	4.42 (2.98)	2.14 (1.46)	0.017*
**Unusual sensory interest**	**Male n. 25**	**Female n. 5**	
ADI-R A	13.88 (6.25)	13.40 (4.04)	0.83
ADI-R B	9.84 (3.25)	10.40 (3.21)	0.73
ADI-R C	4.32 (2.95)	2.80 (1.92)	0.18

**Discussion**.

The results from this study sample show differences in symptomatic patterns between sex/gender in ASD patients. Our results are consistent with an increasing number of publications that have found a different presentation of ASD in males and females (Frazier et al., 2014; Hull et al., 2017). In preschool children with ASD, we did not find any statistically significant sex/gender differences in social interaction or communication, while males appeared slightly more prone to display restricted and repetitive behavior. This finding is in line with Øien and Eisemann (2016), who found that boys were more rigid in behavior and interests than girls.

Studies have reported that the neurobiology of autism is potentially modulated both quantitatively and qualitatively by biological sex (Lai et al., 2017), though it is unclear whether there are specific brain regions or networks that consistently show these modulation effects. A recent systematic review (Mo et al., 2021) reported that despite some well-powered studies that have identified specific patterns of significant sex/gender modulation of autism-control differences, many other studies are likely underpowered, suggesting a critical need for future investigations into sex/gender-based heterogeneity with better powered designs. However, behavioral indicators seem to present differently in autistic males and females and may have different phenotypes (Ecker, 2017), therefore explicit investigations into ASD phenotypes across sexes/genders are recommended.

Recent interest in the ‘female autism phenotype’ (Lai et al., 2015) stems from autobiographical writings of lived experiences, clinical observations, and emerging qualitative research with autistic girls and women, particularly those diagnosed later in life (Bargiela, Steward, & Mandy, 2016). Diagnosed girls seem more likely to engage in reciprocal conversations, share interests, integrate nonverbal and verbal expressive behaviors, show better imagination, initiate friendships, and display less stereotyped use of objects and less narrow interests (Hiller et al., 2014). Regarding developmental change, girls diagnosed at a young age were more likely to have better cognitive development, less intense autistic symptoms, and a reduction in symptoms over time (Lai & Szatmari, 2020).

The present research did not find significant sex/gender differences in terms of social behavior (p < 0.99) or communication and language abilities (p < 0.62), in line with some studies showing that both sexes/genders commonly present deficits in this developmental area (Head et al., 2014; Hiller et al., 2014; Rynkiewicz et al., 2016). However, when analyzing different ADI-R scores across variables, the present study revealed a difference in social behavior between males (mean score: 18.45) and females (mean score: 12.67), which was close to statistical significance (p = 0.05) in non-verbal children with ASD. The lack of statistical significance was probably due to the small sample size, though our findings are consistent with other studies (Hartley and Sikora, 2009; Lawson et al., 2018).

Instead, a strong statistical significance (p < 0.01) was found in restricted and repetitive behaviors between males (mean score: 4.36) and females (mean score: 2.42) as measured by the ADI-R. This finding is in line with Mandy and colleagues (Mandy et al., 2012). The hypothesis that females might be less prone to restricted and repetitive behaviors has been noted in many studies (Duvekot et al., 2017; Frazier et al., 2014; Rubenstein et al., 2015). Possible explanations for this difference range from having different fixations not easily recognized as ASD-related (Hiller et al., 2014) to an increased ability to mask these signs in females with or without mild cognitive deficits, to genetic variants or environmental factors (Van Wijngaarden-Cremers et al., 2014). However, our study population was homogeneous with respect to cognitive level. This allowed us to investigate sex/gender differences in relation to different groups, such as verbal ability, echolalia, and unusual sensory interest sub-groups. As reported in Tables 2 and 4, significant differences in restricted and repetitive behaviors were found in children with no unusual sensory interests (p < 0.05) and in children with minimal verbal abilities (p < 0.05). These differences are in contrast to Harrop and colleagues (Harrop et al., 2015), who found that girls and boys under the age of 5 were more similar with respect to this core deficit, while they found a trend toward gender-differential growth trajectories. However, different studies highlight that compared to autistic males, females tend to show reduced restricted, repetitive, and stereotyped behaviors and interests, but more self-injurious and compulsive behaviors and sensory challenge (McFayden et al., 2020; Moseley, Hitchiner, & Kirkby, 2018). A lack of difference across social-communication-interaction, cognitive, and adaptive functioning was found in minimally verbal individuals (Howe et al., 2015) and in toddlers/preschoolers diagnosed early in life (Matheis et al., 2019; Duvall et al., 2020).

Literature on possible correlations between unusual sensory interests and restricted and repetitive behaviors between boys and girls is not supported by robust evidence. Future research should try to identify how and in which specific areas restricted and repetitive behaviors differ between females and males with ASD, since it is important to have precise diagnostic assessment tools in order to avoid or reduce mis/underdiagnosis in girls and women.

**Study limitations**.

This study provides information that may increase our understanding of ASD presentation across sexes/genders, though it also has some limitations. First, the relatively small sample size did not allow us to obtain robust evidence. Furthermore, despite no significant differences in the sample population, male and female samples were skewed, with the female sample being much smaller, leading to fewer observations and lower ADI-R administration for females. Therefore further researches with larger samples are needed through systematic study and further questions should be addresses in the broader empirical literature.

**Conclusions**.

A multifaceted approach to understanding differences across sexes/genders in ASD is essential for timely and accurate diagnosis and support. Despite some limitations, the present investigation highlighted differences in the phenotypical presentation of ASD. Preschool children with minimal verbal ability and with no unusual sensory interests showed differences in restricted and repetitive behaviors. Females were less prone to restricted and repetitive behaviors, which poses important challenges to the early identification of children with ASD.

## References

[CR1] American Psychiatric Association (2013). Diagnostic and Statistical Manual of Mental Disorders, Fifth Edition, DSM-5. Arlington, VA

[CR2] Auletta AF, Cupellaro S, Abbate L, Aiello E, Cornacchia P, Norcia C, Sogos C (2020). SCORS-G and card pull effect of TAT stories: A study with a nonclinical sample of children. Assessment.

[CR4] Bargiela S, Steward R, Mandy W (2016). The experiences of late-diagnosed women with autism spectrum conditions: An investigation of the female autism phenotype. Journal of autism and developmental disorders.

[CR5] Berardi, A., Panuccio, F., Pilli, L., Tofan, M., i, Valente, D., & Galeoto, G. (2021). Evaluation instruments for executive functions in children and adolescents: A systematic review. *Expert Review of Pharmacoeconomics & Outcomes Research*, *21*(5), 885–896. 10.1080/14737167.2021.1908889 10.1080/14737167.2021.1908889 33760678

[CR6] Catino E, Di Trani M, Giovannone F, Manti F, Nunziata L, Piccari F, Sogos C (2017). screening for Developmental Disorders in 3-and 4-Year-Old italian children: a Preliminary study. Frontiers in pediatrics.

[CR7] Cavalli G, Galeoto G, Sogos C, Berardi A, Tofani M (2022). The efficacy of executive Function interventions in children with autism spectrum disorder: A systematic review and meta-analysis. Expert review of neurotherapeutics.

[CR8] Duvall, S. W., Huang-Storms, L., Presmanes Hill, A., Myers, J., & Fombonne, E. (2020). No sex differences in cognitive ability in young children with autism spectrum disorder. *Journal of Autism & Developmental Disorders*, *50*(5), 1770–1785. 10.1007/s10803-019-03933-1 30810843

[CR9] Duvekot, J., van der Ende, J., Verhulst, F. C., Slappendel, G., van Daalen, E., Maras, A., & Greaves-Lord, K. (2017). Factors influencing the probability of a diagnosis of autism spectrum disorder in girls versus boys. *Autism*, *21*(6), 646–658. 10.1177/1362361316672178 10.1177/1362361316672178 27940569

[CR10] Dworzynski K, Ronald A, Bolton P, Happé F (2012). How different are girls and boys above and below the diagnostic threshold for autism spectrum disorders?. Journal of the American Academy of Child & Adolescent Psychiatry.

[CR11] Ecker C (2017). The neuroanatomy of autism spectrum disorder: An overview of structural neuroimaging findings and their translatability to the clinical setting. Autism.

[CR12] Fioriello F, Maugeri A, D’Alvia L, Pittella E, Piuzzi E, Rizzuto E, Del Prete Z, Manti F, Sogos C (2020). A wearable heart rate measurement device for children with autism spectrum disorder. Scientific Reports.

[CR13] Frazier TW, Georgiades S, Bishop SL, Hardan AY (2014). Behavioral and cognitive characteristics of females and males with Autism in the Simons Simplex collection. Journal of American Academy of Child and Adolescent Psychiatry.

[CR14] Gould, J. (2017). Towards understanding the under-recognition of girls and women on the autism spectrum. *Autism*, *21*(6), 703–705. 10.1177/1362361317706174 10.1177/1362361317706174 28749237

[CR15] Harrop C, Gulsrud A, Kasari C (2015). Does gender moderate core deficits in ASD? An investigation into restricted and repetitive behaviors in girls and boys with ASD. Journal of autism and developmental disorders.

[CR16] Hartley SL, Sikora DM (2009). Sex differences in autism spectrum disorder: an examination of developmental functioning, autistic symptoms, and coexisting behavior problems in toddlers. Journal of Autism and Developmental Disorders.

[CR17] Head, A. M., McGillivray, J. A., & Stokes, M. A. (2014). Gender differences in emotionality and sociability in children with autism spectrum disorders.Mol Autism., volume5 pp.19 10.1186/2040-2392-5-19 PMC3945617 24576331

[CR18] Hiller RM, Young RL, Weber N (2014). Sex differences in autism spectrum disorder based on DSM-5 criteria: evidence from clinician and teacher reporting. Journal of Abnormal Child Psychology. Volume.

[CR19] Howe YJ, O’Rourke JA, Yatchmink Y, Viscidi EW, Jones RN, Morrow EM (2015). Female autism phenotypes investigated at different levels of language and developmental abilities. Journal of autism and developmental disorders.

[CR20] Hull, L., Mandy, W., & Petrides, K. V. (2017). *Behavioural and cognitive sex/gender differences in autism spectrum condition and typically developing males and females* (21 vol., pp. 706–727). Autism. 6 10.1177/1362361316669087 28749232

[CR21] Kanner L (1946). Irrelevant and metaphorical language in early infantile autism. American Journal of Psychiatry.

[CR22] Kim, Y. S., Leventhal, B. L., Koh, Y. J., et al. (2011). Prevalence of autism spectrum disorders in a total population sample. Am J Psychiatry, 168 (2011), pp. 904–912 10.1176/appi.ajp.2011.10101532 21558103

[CR23] Lai MC, Lombardo MV, Baron-Cohen S (2014). Autism. Lancet.

[CR24] Lai MC, Szatmari P (2020). Sex and gender impacts on the behavioural presentation and recognition of autism. Current Opinion in Psychiatry.

[CR25] Lai MC, Lerch JP, Floris DL, Ruigrok AN, Pohl A, Lombardo MV, Baron-Cohen S (2017). Imaging sex/gender and autism in the brain: Etiological implications. Journal of neuroscience research.

[CR26] Lai MC, Lombardo MV, Auyeung B, Chakrabarti B, Baron-Cohen S (2015). Sex/gender differences and autism: setting the scene for future research. Journal of the American Academy of Child & Adolescent Psychiatry.

[CR28] Lawson LP, Joshi R, Barbaro J (2018). Gender Differences During Toddlerhood in Autism Spectrum Disorder: A Prospective Community-Based Longitudinal Follow-Up Study. Journal of Autism and Developmental Disorders.

[CR29] Lord C, Rutter M, Le Couteur A (1994). Autism Diagnostic InterviewRevised: A revised version of a diagnostic interview for caregivers of individuals with possible pervasive developmental disorders. Journal of Autism and Developmental Disorders.

[CR31] Mandy W, Chilvers R, Chowdhury U, Salter G, Seigal A, Skuse D (2012). Sex differences in autism spectrum disorder: Evidence from a large sample of children and adolescents. Journal of Autism and Developmental Disorders. Volume.

[CR32] Manti F, Giovannone F, Sogos C (2019). Parental stress of preschool children with generalized anxiety or oppositional defiant disorder. Frontiers in pediatrics.

[CR33] Matheis M, Matson JL, Hong E, Cervantes PE (2019). Gender differences and similarities: Autism symptomatology and developmental functioning in young children. Journal of autism and developmental disorders.

[CR34] Mattila, M. L., Kielinen, M., Linna, S. L., et al. (2011). Autism spectrum disorders according to DSM-IV-TR and comparison with DSM-5 draft criteria: an epidemiological study. J Am Acad Child Adolesc Psychiatry, 50 (2011), pp. 583–592 10.1016/j.jaac.2011.04.001 21621142

[CR35] McFayden TC, Antezana L, Albright J, Muskett A, Scarpa A (2020). Sex differences in an autism spectrum disorder diagnosis: Are restricted repetitive behaviors and interests the key?. Review Journal of Autism and Developmental Disorders.

[CR36] Milner V, McIntosh H, Colvert E, Happé F (2019). A Qualitative Exploration of the Female Experience of Autism Spectrum Disorder (ASD). Journal of Autism and Developmental Disorders.

[CR37] Mo K, Sadoway T, Bonato S, Ameis SH, Anagnostou E, Lerch JP, Lai MC (2021). Sex/gender differences in the human autistic brains: A systematic review of 20 years of neuroimaging research. NeuroImage: Clinical.

[CR38] Moseley RL, Hitchiner R, Kirkby JA (2018). Self-reported sex differences in high-functioning adults with autism: a meta-analysis. Molecular autism.

[CR39] Øien RA, Hart L, Schjølberg S, Wall CA, Kim ES, Nordahl-Hansen A, Shic F (2017). Parent-endorsed sex differences in toddlers with and without ASD: Utilizing the M-CHAT. Journal of autism and developmental disorders.

[CR40] Øien R, Eisemann MR (2016). Brief report: Parent-reported problems related to communication, behavior and interests in children with autistic disorder and their impact on quality of life. Journal of autism and developmental disorders.

[CR41] Robinson, E. B., Lichtenstein, P., Anckarsäter, H., Happé, F., & Ronald, A. (2013). Examining and interpreting the female protective effect against autistic behavior. Proceedings of the National Academy of Sciences, 110(13), 5258–5262 10.1073/pnas.1211070110 PMC3612665 23431162

[CR42] Ros-Demarize, R., Bradley, C., Kanne, S. M., Warren, Z., Boan, A., Lajonchere, C., Park, J., & Carpenter, L. A. (2020). ASD symptoms in toddlers and preschoolers: An examination of sex differences. *Autism Research: Official Journal of the International Society for Autism Research*, *13*(1), 157–166. 10.1002/aur.2241 10.1002/aur.2241 31747131

[CR43] Rubenstein E, Wiggins LD, Li-Ching Lee LC (2015). A Review of the Differences in Developmental, Psychiatric, and Medical Endophenotypes Between Males and Females with Autism Spectrum Disorder. Journal of Developmental Physical Disabilities Volume.

[CR44] Rutter, M., Le Couteur, A., & Lord, C. (2003). Autism Diagnostic Interview Revised (ADI-R). Los Angeles, CA: Western Psychological Services. Italian edition by R. Faggioli, M. Saccani, A.M. Persico, R. Tancredi, B. Parrini e R. Igliozzi (2005); Giunti Psychometrics

[CR45] Rynkiewicz, A., Schuller, B., Marchi, E., et al. (2016). *An investigation of the ‘female camouflage effect’ in autism using a computerized ADOS-2 and a test of sex/gender differences* (7 vol., p. 10). Molecular Autism 10.1186/s13229-016-0073-0 PMC4721191 26798446

[CR46] Salomone E, Charman T, McConachie H, Warreyn P (2016). Child’s verbal ability and gender are associated with age at diagnosis in a sample of young children with ASD in Europe. Child: Care, Health and Development.

[CR47] Skuse D, Warrington R, Bishop D, Chowdhury U, Lau J, Mandy W, Place M (2004). The developmental, dimensional and diagnostic interview: A novel computerized assessment for autism spectrum disorders. Journal of the American Academy of Child and Adolescent Psychiatry. Volume.

[CR48] Szatmari P, Liu XQ, Goldberg J (2012). Sex differences in repetitive stereotyped behaviors in autism: Implications for genetic liability. American Journal of Medical Genetics. Part B, Neuropsychiatric Genetics.

[CR49] Tofani M, Galeoto G, Cazzetta D, Berardi A, Sansoni J, Valente D (2019). Validation of the pediatric evaluation of disability inventory in an Italian population with autism spectrum disorder: a cross-sectional study. La Clinica Terapeutica.

[CR50] Van Wijngaarden-Cremers PJM, van Eeten E, Groen WB, Van Deurzen PA, Oosterling IJ, Van der Gaag RJ (2014). Gender and age differences in the core triad of impairments in autism spectrum disorders: a systematic review and meta-analysis. Journal of Autism and Developmental Disorders.

[CR51] World Health Organization (2021). Gender and Health. Available at http://www.who.int/gender/whatisgender/en/. Accessed 14 Dec, 2021

